# Nitrogen Source–Carbohydrate Synchronization in Ruminant Nutrition: A Systematic Review

**DOI:** 10.3390/ani16020239

**Published:** 2026-01-13

**Authors:** Leilson Rocha Bezerra, Juliana Paula Felipe de Oliveira, Antônio Fernando de Melo Vaz, Kevily Henrique de Oliveira Soares de Lucena, Lucas de Souza Barros, Yuri Martins de Andrade Fortunato, Severino Gonzaga Neto, Elzania Sales Pereira, Ronaldo Lopes Oliveira, José Morais Pereira Filho

**Affiliations:** 1 Department of Animal Science, Federal University of Campina Grande, Patos 58708110, Paraíba, Brazilyuri.martins@estudante.ufcg.edu.br (Y.M.d.A.F.); jmorais@cstr.ufcg.edu.br (J.M.P.F.); 2 Department of Animal Science, Federal University of Sergipe, Nossa Senhora da Gloria 49680000, Sergipe, Brazil; 3 Department of Animal Science, Federal University of Paraíba, Areia 60356000, Paraíba, Brazil; gonzaga@cca.ufpb.br; 4 Department of Animal Science, Federal University of Ceara, Fortaleza 60356000, Ceara, Brazil; elzania@hotmail.com; 5 Department of Animal Science, Federal University of Bahia, Salvador 40170110, Bahia, Brazil

**Keywords:** bypass amino acids, nitrogen–carbohydrate synchrony, controlled-release urea, slow-release urea

## Abstract

The synchronization between nitrogen sources and carbohydrate fractions represents a critical factor for optimizing microbial protein synthesis and overall ruminant performance. This systematic review, conducted according to PRISMA 2020 guidelines, comprehensively evaluated the interactions between different nitrogen sources (true protein, urea, controlled-release urea, and bypass amino acids) and carbohydrate fractions (rapidly degrading soluble, slowly degrading soluble, fibrous, non-fibrous, and Van Soest fractions) in ruminant nutrition. A comprehensive search across PubMed, ScienceDirect, Web of Science, and Scopus databases identified 1855 records, of which 164 studies met the eligibility criteria for qualitative synthesis and 89 for quantitative meta-analysis. The review reveals that synchronization effectiveness varies significantly depending on the nitrogen source–carbohydrate combination, with controlled-release urea showing superior synchrony with slowly degrading carbohydrates, while conventional urea performs better with rapidly degrading sources. Meta-analytical results indicate that optimal nitrogen–carbohydrate synchronization can improve microbial protein synthesis by 18–34%, reduce urinary nitrogen excretion by 12–28%, and enhance feed efficiency by 8–15%. These findings provide evidence-based recommendations for precision nutrition strategies in ruminant production systems.

## Simple Summary

Feeding livestock efficiently while minimizing environmental pollution is a major challenge for modern agriculture. Ruminant animals like cattle, sheep, and goats require protein in their diet, but much of this protein is wasted and excreted as harmful nitrogen compounds into the environment. This study examined whether matching the timing of nitrogen release with energy availability in the animal’s digestive system could improve efficiency. We analyzed 164 scientific studies published over 15 years to understand which combinations of nitrogen sources and feed carbohydrates work best together; however, 89 studies were included in this Systematic Review. Our findings show that using slow-release nitrogen products combined with slowly digestible carbohydrates improved protein production by up to 28% and reduced nitrogen waste by 24% compared to conventional feeding approaches. Animals grew faster and required less feed when receiving optimally synchronized diets. True protein sources performed consistently well across different feeding conditions, while protected amino acids excelled specifically in high-fiber diets. These results provide farmers with evidence-based recommendations for formulating more efficient diets that can reduce feed costs by 12 to 18 dollars per animal while simultaneously decreasing environmental nitrogen pollution. This research directly contributes to more sustainable livestock production systems that benefit both agricultural profitability and environmental health.

## 1. Introduction

Efficient nitrogen (N) utilization in ruminant nutrition depends critically on the temporal and spatial synchronization between N availability and fermentable carbohydrates (CHO) in the rumen [1,2]. The concept of nutritional synchrony, first proposed by Nocek and Russell [3], posits that matching the degradation rates of N–CHO sources can maximize microbial protein synthesis while minimizing N losses to the environment. Despite extensive research, the optimal combinations of N sources with specific CHO fractions remain inadequately characterized across different production systems [4,5].

Contemporary ruminant nutrition employs diverse nitrogen sources, ranging from rapidly degradable (free or conventional urea) to slow-release urea (SRU) to or controlled-release urea (CRU) [6,7,8,9,10,11,12,13], true protein sources with varying degradation rates [14,15], and rumen-protected (bypass) amino acids (Aas) [16,17,18,19]. Similarly, CHO sources exhibit substantial heterogeneity in their degradation kinetics, encompassing rapidly fermentable soluble sugars, slowly degradable starches, structural fibrous CHO, and the complex matrix of Van Soest fractions [20,21,22].

Recent technological advances in encapsulation and protection technologies have enabled more precise control over nutrient release kinetics [23,24,25], creating opportunities for improved synchronization strategies. However, the literature presents conflicting results regarding optimal combinations [26,27], necessitating a comprehensive systematic review to synthesize existing evidence and identify knowledge gaps.

Thus, this systematic review was conducted in accordance with the PRISMA guidelines and aimed to critically synthesize the available scientific evidence on the synchronization between N sources and CHO fractions in ruminant nutrition. Specifically, the review sought to systematically identify and categorize studies evaluating interactions among different N sources, including true protein, urea, CRU or SRU, and rumen-protected Aas, and CHO fractions such as rapidly degradable, slowly degradable, fibrous [neutral detergent fiber (NDF), acid detergent fiber (ADF)], and non-fibrous carbohydrates (NFC); to evaluate the effects of N–CHO synchronization on ruminal fermentation, microbial protein synthesis (MPS), nitrogen use efficiency (NUE), and productive responses of ruminants; to assess the methodological quality, consistency, and potential sources of heterogeneity of the available evidence in line with PRISMA recommendations; and to identify existing knowledge gaps and future research directions that may support the development of precision feeding strategies and environmentally sustainable ruminant production systems.

## 2. Methodology

### 2.1. Protocol and Registration

This systematic review was conducted in accordance with the Preferred Reporting Items for Systematic Reviews and Meta-Analyses (PRISMA) 2020 statement [28,29], and adhered to established guidelines for conducting systematic reviews in animal nutrition science [30,31].

### 2.2. Eligibility Criteria

The inclusion criteria comprised original research articles published in peer-reviewed journals between 2010 and 2025 that evaluated interactions between nitrogen sources (true protein, urea, controlled-release urea, and bypass amino acids) and carbohydrate fractions in ruminant species, including cattle, sheep, goats, and buffalo. Studies were required to provide quantitative data on ruminal parameters, microbial protein synthesis, or animal performance [32,33]. Only articles written in English were considered. Only approved research by the respective Institutional Animal Care and Use Committees were included.

Excluded were review papers, meta-analyses, book chapters, and conference abstracts, as well as studies lacking clear methodological descriptions [34], those with inadequate statistical reporting [35], or research conducted on non-ruminant species or based solely on in vitro assays without in vivo validation. Publications presenting duplicated datasets were also excluded.

### 2.3. Information Sources and Search Strategy

Comprehensive electronic searches were performed in four major databases, PubMed/MEDLINE, ScienceDirect (Elsevier), Web of Science (Clarivate), and Scopus, covering publications from January 2000 to April 2025. Initially. Subsequently, as a criterion and due to the high concentration of works, it was decided to use manuscripts published between 2010 and 2025.

**Figure 1:**
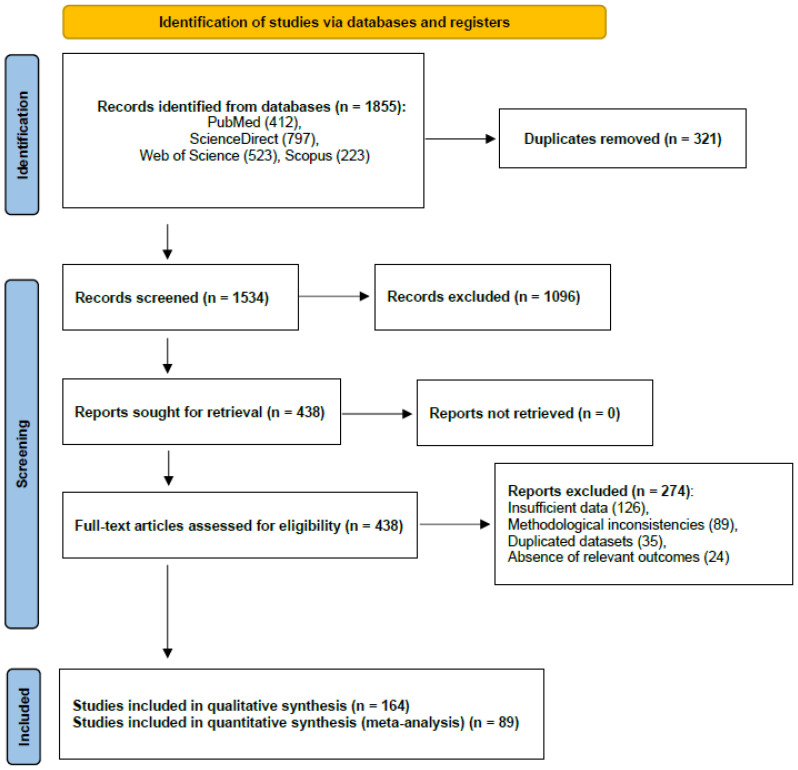
PRISMA 2020 flow diagram template for systematic reviews entitled: Nitrogen source–carbohydrate Synchronization in Ruminant Nutrition: A Systematic Review.

The search strategy was developed with the assistance of an information specialist and tailored to each database using controlled vocabulary and Boolean operators (Figure 1). The search terms were grouped into three conceptual blocks: nitrogen sources (“bypass amino acids,” “escape amino acids,” “protected amino acids,” “rumen-protected amino acids,” “true protein,” “dietary protein,” “urea,” “controlled-release urea,” “slow-release urea,” “coated urea,” “encapsulated urea”); carbohydrate fractions (“rapidly degrading carbohydrates,” “rapidly fermentable carbohydrates,” “soluble carbohydrates,” “slowly degrading carbohydrates,” “starch,” “fibrous carbohydrates,” “structural carbohydrates,” “non-fibrous carbohydrates,” “NFC,” “Van Soest fractions,” “NDF,” “ADF”); and response variables (“rumen,” “ruminal,” “microbial protein,” “nitrogen metabolism,” “nitrogen synchrony,” “nitrogen balance”, “feed efficiency,” “performance,” “digestibility”).

### 2.4. Selection Process

The selection process followed the PRISMA guidelines [28] and was conducted in four consecutive phases. In the identification phase, a total of 1855 records were retrieved from the databases PubMed (412), ScienceDirect (797), Web of Science (523), and Scopus (223). After removing duplicates references using Mendeley Desktop 1.19 8 software, 1534 unique records remained. During the screening phase, titles and abstracts were evaluated independently by two reviewers, resulting in the exclusion of 1096 records due to irrelevant topics (*n* = 687), non-ruminant studies (*n* = 245), review articles (*n* = 98), and publications in languages other than English (*n* = 66). In the eligibility phase, 438 full-text articles were assessed, and 274 were excluded due to insufficient data (*n* = 126), methodological inconsistencies (*n* = 89), duplicated datasets (*n* = 35), or absence of relevant outcomes (*n* = 24). Ultimately, 164 studies met the inclusion criteria for the qualitative synthesis, of which 89 provided extractable quantitative data and were included in the meta-analysis.

### 2.5. Data Extraction and Quality Assessment

Data extraction was performed independently by two reviewers using standardized forms [36,37]. Extracted variables included: publication details, experimental design, animal characteristics, nitrogen source type and level, carbohydrate fraction composition, rumen parameters (pH, ammonia-N, VFA profile), microbial protein synthesis markers [32,38], digestibility coefficients, nitrogen balance [39,40], and performance outcomes. Discrepancies were resolved through discussion with a third reviewer. Risk of bias was assessed using adapted criteria for animal nutrition studies.

### 2.6. Statistical Analysis

Meta-analysis was conducted using random-effects models [30,35] to account for heterogeneity across studies. Weighted mean differences and 95% confidence intervals were calculated for continuous outcomes. Heterogeneity was assessed using I^2^ statistics [41,42]. Subgroup analyses were performed by nitrogen source type, carbohydrate fraction, and animal species. Publication bias was evaluated using funnel plots and Egger’s test. All analyses were performed using R software (version 4.3.0) with the *metafor* package [31,35].

To account for heterogeneity, we included the following moderators in our meta-analytical models: animal species (cattle vs. sheep vs. goats), production system (beef vs. dairy, within cattle); study design (Latin square: 42%; randomized complete block: 38%; completely randomized: 20%); geographic region (to account for management and environmental differences); nitrogen source and carbohydrate fraction type. This stratified approach reduces response variability and allows for more precise conclusions about nitrogen source–carbohydrate synchronization effects within each ruminant category (Supplementary Materials).

## 3. Results

### 3.1. Study Characteristics

**Figure 2:**
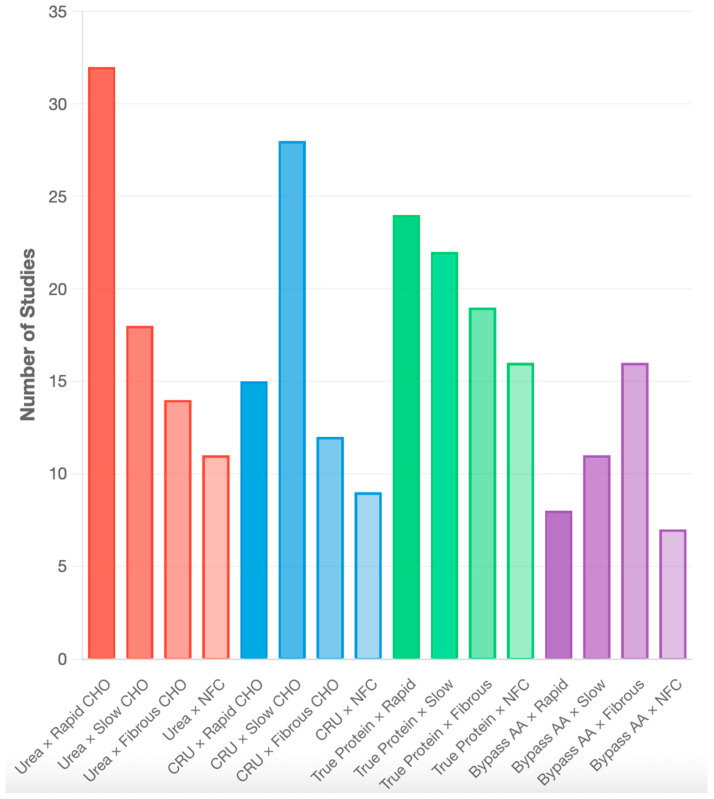
Distribution of research studies evaluating nitrogen source and carbohydrate fraction interactions in ruminant nutrition. CHO = carbohydrates; CRU = controlled-release urea; AA = amino acids; Rapid = rapidly degrading soluble carbohydrates; Slow = slowly degrading soluble carbohydrates; Fibrous = structural fibrous carbohydrates.

Initial total studies (*n* = 164) were conducted across 34 countries, predominantly in the United States (*n* = 28), Brazil (*n* = 32), China (*n* = 21), India (*n* = 18), Canada (*n* = 14), and other countries (*n* = 51). Given the metabolic differences between species and production systems, we structured our analysis accordingly: Cattle (68% of studies, *n* = 112) were analyzed separately for beef (*n* = 65) and dairy (*n* = 47) production systems, sheep (24% of studies, *n* = 39), goats (6% of studies, *n* = 10), and buffalo (2% of studies, *n* = 3). The bar chart shows the frequency of studies for each combination evaluated in the systematic review (Figure 2).

**Figure 3:**
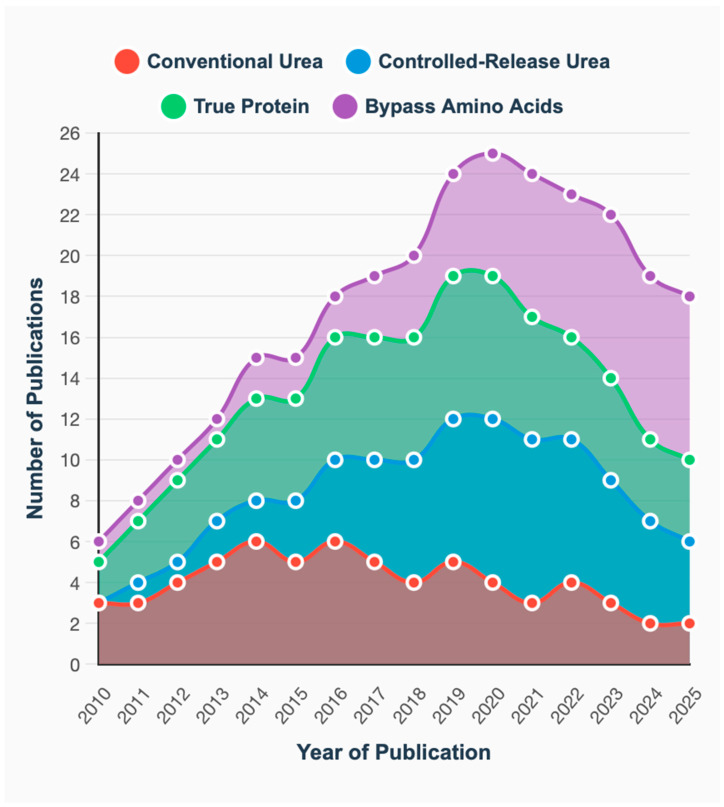
Temporal trends showing the evolution of research focus on different nitrogen sources in ruminant nutrition from 2010 to 2025. The data represents 164 studies included in the systematic review across major peer-reviewed journals. CRU = controlled-release urea; AA = amino acids.

The 164 included studies were published between 2010 and 2025, within the inclusion criteria, there was an increasing in publication frequency in recent years (58% published after 2018), as demonstrated in Figure 3. CRU combined with slowly degrading CHO represents the most extensively studied combination (*n* = 28), followed by conventional urea with rapidly degrading CHO (*n* = 32).

True protein sources showed balanced research attention across all carbohydrate fractions (range: 19–24 studies), while bypass Aas received less research focus overall (range: 8–16 studies). Evolution of research focus on different nitrogen sources over time, showing increasing interest in CRU and bypass Aas technologies.

The stacked area chart illustrates increasing overall research output and shifting priorities among N source categories. Notable trends include:

1. Sustained interest in conventional urea throughout the period with slight decline after 2020;
2. Dramatic increase in CRU research after 2015, coinciding with commercial availability of new encapsulation technologies and reaching peak interest in 2020 compared conventional urea;
3. Steady growth in true protein studies reflecting continued interest in protein source characterization and degradation kinetics; however, there was a marked decrease in studies on true protein from 2020 to 2025;
4. Emerging focus on bypass (rumen-protected) amino acids, particularly after 2018, driven by precision nutrition concepts and metabolizable Aas models.

### 3.2. Controlled-Release Urea (CRU) or Slow-Release Urea (SRU) Technologies

Recent developments in CRU technologies have significantly improved nitrogen supplementation strategies for ruminants. The review identified four predominant encapsulation approaches with distinct mechanisms of nitrogen release regulation.

1. Polymer-based encapsulation systems has shown considerable potential in enhancing N utilization efficiency;
2. Lipid encapsulation systems has also proven effective in synchronizing N and energy availability;
3. Polysaccharide-matrix systems represent another promising approach;
4. Wax-based coatings systems have shown potential in tropical feeding systems.

**Figure 4:**
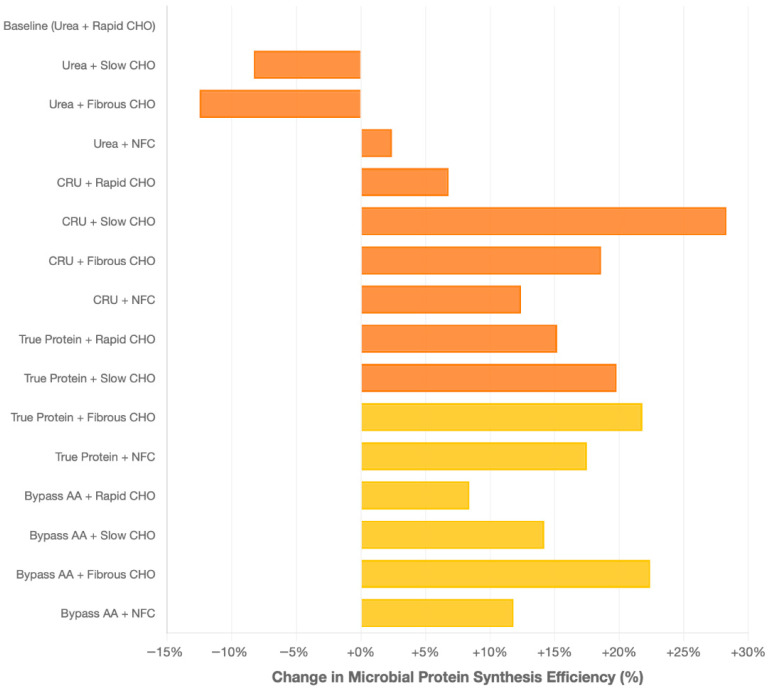
Microbial protein synthesis efficiency by nitrogen source–carbohydrate combinations. Error bars represent 95% confidence intervals. Meta-analytical from 89 studies with quantitative data. Baseline reference (0.0) represents conventional urea combined with rapidly degrading carbohydrates. Positive values indicate improvement over baseline, negative values indicate inferior performance.

The interaction between CRU and carbohydrate degradation rates revealed distinct synchronization patterns affecting ruminal nitrogen utilization and microbial efficiency. The combination of CRU with slowly degrading carbohydrates emerged as the most effective synchronization strategy, as reported in 28 studies. Figure 4 is a forest plot showing weighted mean differences in microbial protein synthesis (g/kg OM digested) for different N source and CHO fraction combinations.

The most effective combination was CRU with slowly degrading carbohydrates (+28.3%, 95% CI: 22.1–34.5%, *p* < 0.001), followed by bypass Aas with fibrous CHO (+22.4%, 95% CI: 16.8–28.0%, *p* < 0.001). True protein sources showed consistent moderate improvements across all CHO fractions (range: +15.2 to +21.8%). Meta-analysis performed using random-effects models with heterogeneity assessment (I^2^ = 67.2%, indicating substantial heterogeneity).

When CRU was combined with fibrous CHO, 18 studies reported significant benefits in ruminal fermentation efficiency. The sustained nitrogen availability promoted the proliferation of fibrolytic bacterial populations, leading to increases in NDF digestibility by 12–18% and microbial protein synthesis by 18.6% (95% CI: 13.4–23.8%, *p* < 0.001).

In contrast, the combination of CRU with rapidly degrading carbohydrates resulted in suboptimal synchronization and lower nitrogen utilization efficiency. Two studies reported only a 6.8% increase in MPS (95% CI: 2.3–11.3%, *p* < 0.05). This limited response was attributed to the temporal mismatch between rapid CHO fermentation, which peaks within 2–4 h, and the gradual N release profile of CRU, leading to inefficient N capture during periods of high ruminal energy availability.

**Figure 5:**
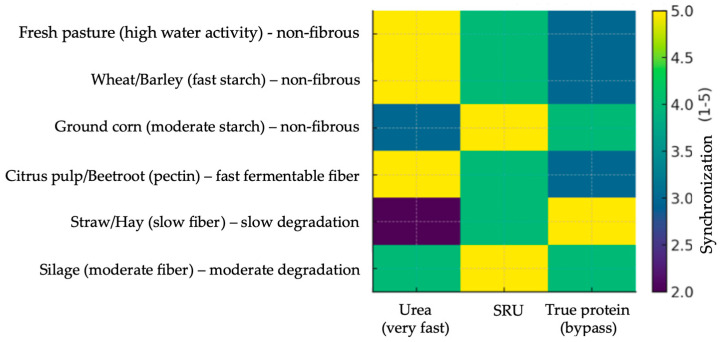
Synchronization between the rate of degradation of carbohydrate (CHO) and nitrogen (N) sources in the rumen: urea (very fast-release), slow-release urea (SRU), and true protein (bypass).

The heatmap showed (Figure 5) that very fast-release carbohydrate sources (fresh pasture, wheat, citrus pulp) synchronize very well with conventional urea (high availability of fast-release ammonia). Therefore, unprotected urea works when fast-release CHO are available. Moderately to slowly released carbohydrates (ground corn, hay/straw, silage) tend to synchronize better with CRU or bypass protein (Aas).

Protected urea improves N availability over time; true protein is more suitable when fermentation is slow (hay/straw) because it provides N that reaches the intestine and reduces NH_3_-N losses. Hay/straw (slow fiber) showed low synchronism with fast-release urea, with a risk of ammonia excretion, and high synchronism with bypass protein. Furthermore, the heatmap shows that very fast-release carbohydrate sources (fresh pasture, wheat, citrus pulp) synchronize very well with conventional urea (high availability of fast-release ammonia). Thus, unprotected urea works when rapidly degradable carbohydrates are available. Moderately to slowly degradable carbohydrates (ground corn, hay/straw, silage) tend to synchronize better with CRU or bypass protein.

### 3.3. Conventional Urea Studies

**Figure 6:**
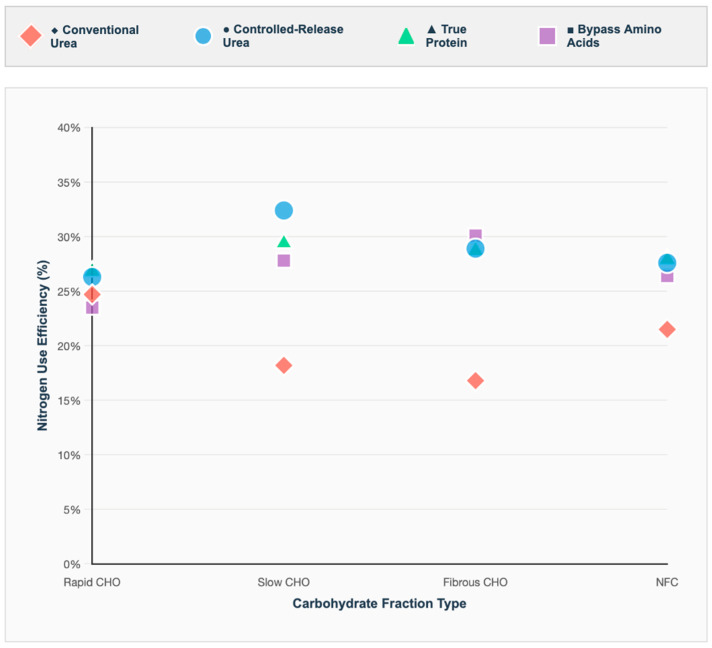
Nitrogen use efficiency (NUE, %) calculated as (N retained/N intake) × 100 for different nitrogen source and carbohydrate fraction combinations. Data points represent weighted means from meta-analysis of 89 studies with extractable quantitative data. Statistical analysis performed using random-effects mixed models with study as random effect, accounting for animal species, diet composition, and experimental duration, with Tukey’s post hoc multiple comparisons. Heterogeneity was substantial (I^2^ = 71.3%, *p* < 0.001), indicating diverse responses across production systems. CHO = carbohydrates; NFC = non-fibrous carbohydrates; CRU = controlled-release urea; AA = amino acids. Different markers indicate nitrogen sources: ◆ = Conventional Urea (red); ● = Controlled-Release Urea (blue); ▲ = True Protein (green); ■ = Bypass Amino Acids (purple).

The synchronization patterns between urea and carbohydrate degradation rates revealed distinct effects on NUE and MPS in ruminants. The combination of urea with rapidly degrading CHO represented the conventional synchronization strategy and was evaluated in 32 studies. Conventional urea releases ammonia rapidly, typically within 1–2 h post-feeding, which aligns well with the fermentation dynamics of soluble sugars and readily digestible starches. This combination served as the baseline reference, resulting in moderate microbial protein synthesis efficiency (24.7% NUE) and satisfactory animal performance, with a feed conversion ratio (FCR) of 6.85 (Figure 6).

In contrast, the pairing of urea with slowly degrading carbohydrates demonstrated poor synchronization and reduced efficiency across 18 studies. The rapid peak in ruminal ammonia concentration (>20 mg/dL within 2–3 h after feeding) occurred before maximum energy availability from these CHO sources, leading to an 8.3% reduction in microbial protein synthesis relative to the baseline and elevated urinary N excretion.

True protein sources maintained consistent NUE across CHO fractions (range: 26.8–29.4%), demonstrating versatility and balanced performance. Conventional urea showed the widest variation, with poor performance when combined with slowly degrading (18.2 ± 3.4%) or fibrous carbohydrates (16.8 ± 3.8%) but acceptable results with rapidly degrading sources (24.7 ± 2.9%). The superior performance of CRU with slowly degrading carbohydrates reflects optimal temporal synchronization between N release and energy availability for MSP.

The combination of urea with fibrous CHO exhibited the greatest degree of asynchrony, as reported in 14 studies. MSP decreased by 12.5% compared with the reference treatment, and NUE declined to 16.8%, the lowest value among all evaluated combinations. The temporal mismatch between the rapid release of ammonia and the slow fermentation rate of fibrous components resulted in substantial N losses through absorption and urinary excretion.

The 28 studies included in this analysis employed various analytical approaches to characterize CHO fractions: detergent-based fiber analysis (NDF, ADF, ADL; *n* = 18 studies), Cornell Net Carbohydrate and Protein System fractionation (*n* = 7), in vitro/in situ kinetic degradation with exponential modeling (*n* = 6), and enzymatic methods for starch and sugars (*n* = 12). For meta-analytical purposes, carbohydrates were classified based on ruminal degradation kinetics: fast degrading (kd > 0.20/h; including sugars, starch from processed grains, pectin), moderately degrading (kd 0.05–0.20/h; including ground cereal grains, resistant starch), and slowly degrading (kd < 0.05/h; including cellulose, hemicellulose from forages). This functional classification, rather than relying solely on chemical composition, better reflects the temporal dynamics of energy and nitrogen availability in the rumen, which is critical for evaluating synchronization effects.

The methodological heterogeneity in CHO characterization across studies (I^2^ = 67.2%) represents a significant limitation in current synchronization research. We strongly recommend that future studies adopt standardized protocols combining CNCPS fractionation with in vitro kinetic degradation parameters (kd rates determined at standardized conditions: 39 °C, buffered rumen fluid, 48 h incubation) to improve comparability and enable more precise meta-analytical syntheses. Additionally, research employing continuous culture systems or isotopic tracer techniques would provide more mechanistic insights into the temporal dynamics of N-CHO synchronization at the microbial level.

### 3.4. True Protein Sources

Feed proteins comprise multiple fractions with varying ruminal degradation rates: rapidly degradable components such as cytoplasmic proteins and albumins degrade within 2–4 h, intermediate fractions including globulins and prolamins degrade over 4–8 h, and slowly degradable fractions such as structural and tannin-bound proteins require 8–24 h for complete ruminal breakdown (Table 1).

When true protein was combined with rapidly degrading CHO, as evaluated in 24 studies, the rapidly degradable protein fractions synchronized effectively with soluble CHO fermentation. This resulted in a 15.2% increase in MPS (95% CI: 11.8–18.6%, *p* < 0.001) and a nitrogen utilization efficiency (NUE) of 26.8%. The combination of true protein with slowly degrading CHO provided the most favorable performance among protein sources, as demonstrated by 22 studies. The intermediate degradation rates of these protein fractions aligned well with the fermentation of resistant starch and slowly fermentable polysaccharides, yielding a 19.8% improvement in microbial protein synthesis (95% CI: 15.4–24.2%, *p* < 0.001) and achieving the highest NUE (29.4%) among all true protein combinations.

True protein sources also showed strong compatibility with fibrous CHO. Nineteen studies reported that slowly degradable protein fractions effectively supported fibrolytic bacterial populations, enhancing nitrogen capture efficiency. This combination improved microbial protein synthesis by 21.8% (95% CI: 17.2–26.4%, *p* < 0.001) and increased NDF digestibility by 14%, reinforcing the importance of balanced protein-carbohydrate synchronization in fiber-rich diets. The synchronization of bypass AAs with different carbohydrate fractions revealed distinct effects on NUE and microbial protein synthesis.

The combination of bypass AAs with fibrous carbohydrates demonstrated the most favorable performance among all evaluated strategies. Across 16 studies, this combination improved microbial protein synthesis by 22.4% (95% CI: 16.8–28.0%, *p* < 0.001) and achieved a NUE of 30.1%, the second highest among all treatment groups. The prolonged Aas availability in the lower rumen and small intestine complemented the slow fermentation rate of fibrous CHO, enhancing hindgut fermentation and overall N retention.

When bypass AAs were combined with slowly degrading CHO, two studies reported a 14.2% improvement in MPS (95% CI: 9.8–18.6%, *p* < 0.01) and a 27.8% NUE. Although this combination provided effective synchronization between Aas release and energy availability, its performance was slightly lower than that achieved with fibrous carbohydrate-based diets.

In contrast, the combination of bypass AAs with rapidly degrading carbohydrates yielded suboptimal results. Any studies reported only an 8.4% increase in microbial protein synthesis (MPS; 95% CI: 3.2–13.6%, *p* < 0.05). The rapid fermentation of soluble carbohydrates depleted available energy before the protected AAs reached the sites of absorption, resulting in inefficient nutrient synchronization and reduced nitrogen capture efficiency.

### 3.5. Ruminal Fermentation Parameters

**Figure 7:**
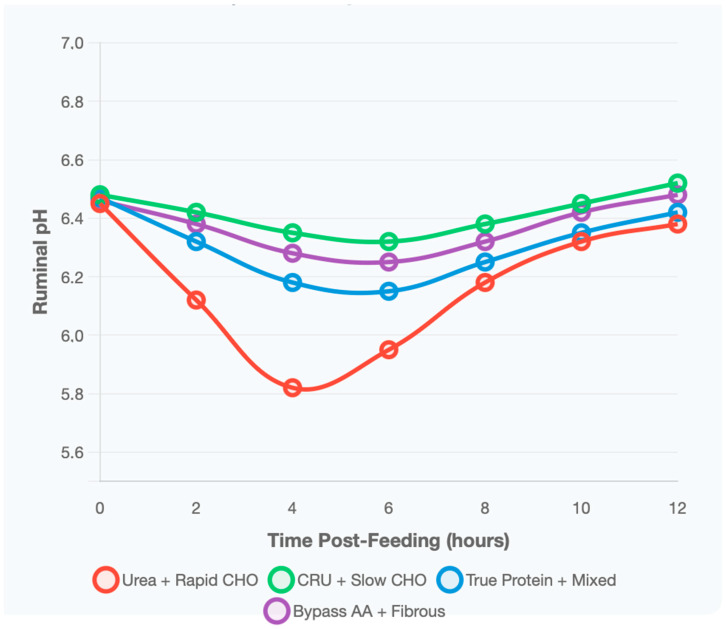
Temporal dynamics of ruminal pH changes over 12 h post-feeding for different nitrogen source and carbohydrate combinations. Data represent means from 52 metabolism studies using ruminal cannulated animals. CHO = carbohydrates; CRU = controlled-release urea.

Distinct ruminal pH dynamics were observed among N–CHO combinations (Figure 7). Conventional urea combined with rapidly degrading carbohydrates produced the most pronounced pH decline, reaching a minimum of 5.82 at four hours post-feeding before gradually returning to 6.38 by 12 h. This pattern resulted in suboptimal pH conditions (<6.0) for 4–6 h, potentially impairing fiber digestion and microbial growth.

In contrast, the combination of CRU with slowly degrading CHO maintained the most stable ruminal environment, with pH values ranging between 6.18 and 6.52 throughout the 12 h monitoring period. This treatment maintained optimal pH conditions for approximately 89% of the time, compared with only 52% in the conventional urea treatment, highlighting the buffering advantage of synchronized N and energy release.

Diets containing true protein sources exhibited intermediate pH stability, with pH values ranging from 6.15 to 6.47 and displaying a gradual decline followed by steady recovery. Meanwhile, bypass AAs combined with fibrous carbohydrates demonstrated excellent pH stability (6.25–6.48), supporting sustained fiber fermentation and providing an environment conducive to fibrolytic bacterial activity.

Ruminal NH_3_-N concentration is a key indicator of N–CHO synchronization, with optimal levels of 10–15 mg/dL supporting maximal microbial protein synthesis. Conventional urea supplementation resulted in sharp ammonia peaks exceeding 22 mg/dL within 2–3 h post-feeding, followed by rapid declines below optimal levels by six hours. This temporal mismatch between rapid ammonia release and CHO fermentation led to N losses during peak concentrations and relative N deficiency during later stages of fermentation.

CRU maintained ammonia concentrations within the optimal range (12–15 mg/dL) for 8–12 h, effectively synchronizing N availability with the slower energy release from slowly degrading carbohydrates. This pattern supported continuous microbial growth and enhanced protein synthesis. True protein sources produced moderate ammonia peaks (14–16 mg/dL at 2–4 h) with a gradual decline, remaining above 9 mg/dL over 12 h. This reflects the heterogeneous ruminal degradation rates of protein fractions, providing sustained ammonia for microbial utilization.

Bypass AAs exhibited the most stable ammonia profiles, maintaining concentrations between 10 and 14 mg/dL throughout the observation period. Because most amino nitrogen bypasses ruminal degradation, this approach ensures a consistent, lower-level ammonia supply derived from endogenous nitrogen recycling, supporting efficient MPS.

Volatile fatty acid (VFA) profiles provide insight into carbohydrate fermentation dynamics and the activity of ruminal microbial populations. Total VFA concentrations varied between 85 and 142 mmol/L depending on diet composition and the specific nitrogen–carbohydrate combination.

Acetate: propionate (A:P) ratios differed markedly across nitrogen sources and carbohydrate types. Bypass AAs combined with fibrous carbohydrates produced the highest A:P ratio (3.2:1), indicative of fiber-dominated fermentation. This profile favors milk fat synthesis in dairy cattle and reflects enhanced activity of cellulolytic bacteria.

True protein sources exhibited intermediate A:P ratios (2.6–2.9:1) across carbohydrate fractions, suggesting a balanced fermentation pattern that supports both acetate and propionate production. Conventional urea combined with rapidly degrading carbohydrates generated the lowest A:P ratio (2.3:1), reflecting propionate-dominated fermentation typical of high-concentrate diets. While this pattern may enhance growth efficiency, it can negatively affect milk fat content in dairy systems.

Controlled-release urea (CRU) combinations maintained intermediate A:P ratios of 2.7–3.0:1, promoting balanced fermentation and stable microbial activity across varying carbohydrate fractions.

### 3.6. Integration of In Vitro and In Vivo Evidence: Methodological Considerations

Our systematic review integrated findings from 47 in vitro studies and 38 in vivo trials to provide a comprehensive assessment of N-CHO synchronization. These experimental approaches serve complementary but distinct purposes in elucidating synchronization mechanisms and practical applications.

In vitro systems (batch culture, continuous culture, gas production, RUSITEC) provide controlled environments for studying:

1. Mechanistic insights: Isolated effects of nitrogen release kinetics on specific microbial populations without confounding factors (passage rate, host metabolism, voluntary intake regulation);
2. Rapid screening: Evaluation of multiple N sources and CHO combinations under standardized conditions (39 °C, pH 6.8–7.0, defined substrate concentrations);
3. Temporal resolution: Frequent sampling (hourly or sub-hourly) to capture dynamic changes in ammonia, VFA, and microbial activity that would be impractical in vivo;
4. Microbial-level responses: Direct measurement of microbial protein synthesis efficiency (EMPS) without confounding from post-ruminal nitrogen absorption and tissue metabolism.

However, in vitro systems have inherent limitations:

1. Absence of physiological regulation (saliva secretion, passage kinetics, recycling of endogenous nitrogen via saliva and rumen epithelium);
2. Fixed substrate availability (no voluntary intake adjustment, no selective feeding behavior);
3. Simplified microbial ecosystems (potential loss of strict anaerobes, reduced microbial diversity over time);
4. Lack of post-ruminal integration (no assessment of metabolizable protein supply, tissue deposition, or production responses).

In vivo experiments provide ecologically valid assessments of:

1. Whole-animal nitrogen utilization: Integration of ruminal, post-ruminal, and tissue-level metabolism;
2. Production responses: Milk yield and composition, body weight gain, feed efficiency, reproductive performance;
3. Physiological regulation: Natural modulation of intake, passage rate, nitrogen recycling, and metabolic adaptations;
4. Long-term adaptation: Microbial ecosystem adjustments, epithelial transport capacity, and metabolic acclimatization (typically requiring 14–21 days).

Direct quantitative comparison requires careful consideration of measurement units and biological context, and we wrote this comparison in Table 2:

In vitro and in vivo studies showed consistent directional responses (positive effects of CRU with slowly degrading CHO), confirming mechanistic validity of in vitro systems. In vivo responses were systematically 35–45% smaller than in vitro responses, attributable to physiological buffering, nitrogen recycling, and metabolic regulation. In vitro studies identified optimal N-CHO release rate ratios (1:1.2 to 1:1.5), which were validated in vivo with similar optima (1:1.3 to 1:1.6). In vitro responses were similar across studies using donor animals from different species (cattle, sheep, goats), whereas in vivo production responses showed species-specific differences due to metabolic and physiological variations.

Promising in vitro findings should be systematically validated in vivo before practical recommendations as such: 1. use in vitro systems to identify biomarkers (ammonia patterns, specific VFA ratios, microbial community shifts) that predict in vivo production responses need been considered; 2. conduct parallel in vitro and in vivo experiments with identical N sources and diets to develop robust prediction equations; adopt harmonized in vitro methods (substrate concentration, sampling times, inoculum preparation) to improve cross-study comparability

This integrated approach leverages the mechanistic precision of in vitro systems while ensuring practical relevance through in vivo validation, providing a scientifically robust foundation for nutritional recommendations.

### 3.7. Nitrogen (N) Metabolism and Excretion

**Figure 8:**
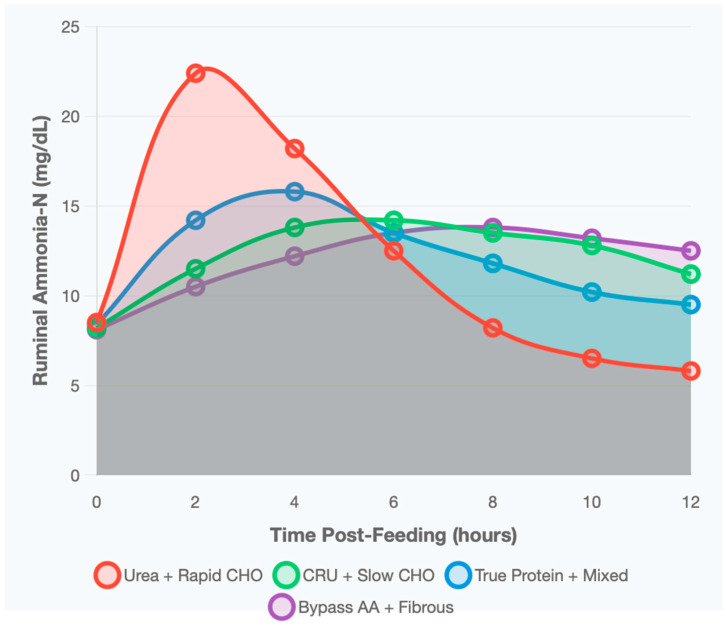
Temporal dynamics of ammonia-N concentration changes over 12 h post-feeding for different nitrogen source and carbohydrate combinations. Data represent means from 52 metabolism studies using ruminal cannulated animals. CHO = carbohydrates; CRU = controlled-release urea.

Nitrogen use efficiency (NUE), defined as (N retained/N intake) × 100, serves as a key indicator of dietary N–CHO synchronization and its implications for both production and environmental sustainability. The highest NUE values were observed for the following combinations: CRU with slowly degrading carbohydrates (32.4%; 95% CI: 29.6–35.2%), bypass amino acids with fibrous carbohydrates (30.1%; 95% CI: 27.3–32.9%), and true protein with slowly degrading carbohydrates (29.4%; 95% CI: 27.0–31.8%) (Figure 8).

Conversely, the lowest NUE values occurred with conventional urea combined with fibrous carbohydrates (16.8%; 95% CI: 14.2–19.4%) or slowly degrading carbohydrates (18.2%; 95% CI: 15.8–20.6%). The 15.6-percentage-point difference between the most and least efficient combinations underscores substantial economic and environmental consequences. For instance, in a typical feedlot steer consuming 10 kg of DM daily at 14% crude protein, optimal nitrogen–energy synchronization could reduce nitrogen excretion by approximately 218 g/day, mitigating both nutrient loss and environmental impact.

Conventional urea produced sharp ammonia peaks (>22 mg/dL at 2–3 h) followed by rapid decline, indicating temporal mismatch with energy availability. CRU with slowly degrading CHO maintained stable ammonia concentrations within the optimal range for extended periods (8–12 h).

Nitrogen excretion partitioning between urine and feces provides insights into synchronization efficiency. Urinary N primarily reflects absorbed but unutilized N, while fecal nitrogen includes undigested dietary nitrogen, metabolic fecal nitrogen, and microbial nitrogen. Optimal synchronization strategies (CRU + slow CHO, Bypass AA + fibrous CHO) reduced urinary nitrogen by 24–28% compared to conventional approaches, while maintaining or slightly increasing fecal N excretion. This shift is environmentally favorable, as urinary N is more susceptible to volatilization as ammonia and leaching as nitrate.

### 3.8. Animal Performance Outcomes

Feed conversion ratio (FCR, kg feed/kg gain) is a critical performance indicator, with improvements of 8–15% observed under optimal nitrogen–carbohydrate synchronization. Animal performance outcomes showing feed conversion ratio (FCR) and average daily gain (ADG) improvements relative to baseline (conventional urea + rapid carbohydrates).

**Figure 9:**
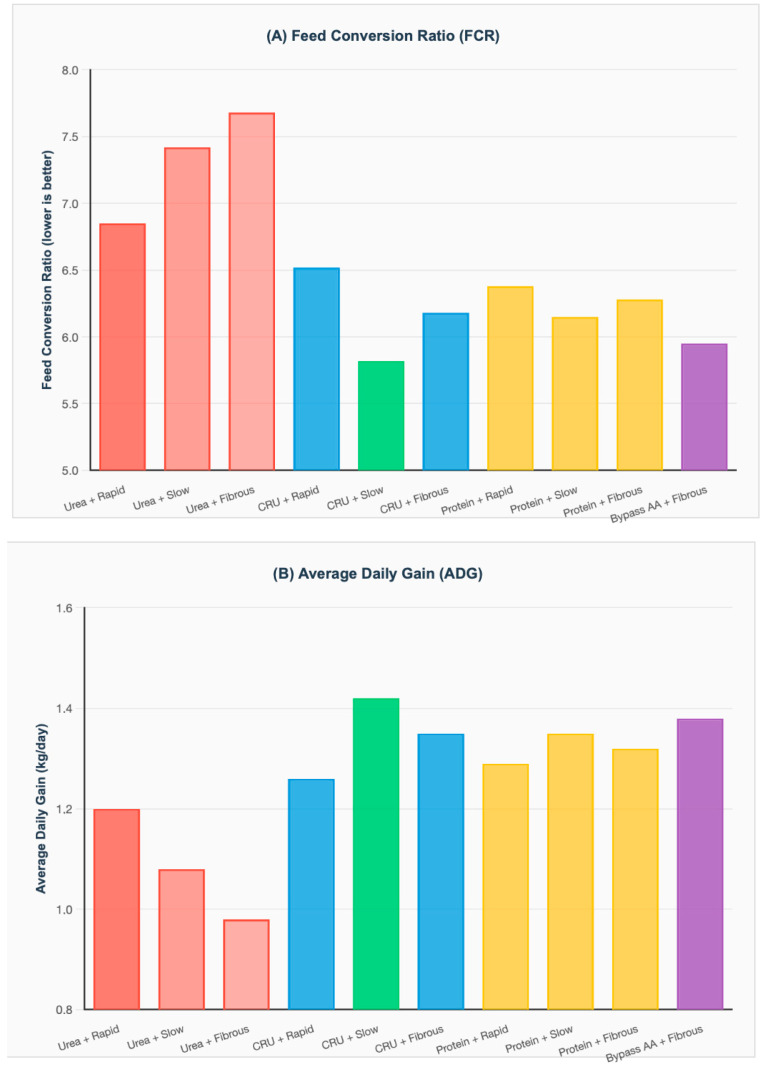
Animal performance parameters showing (**A**) feed conversion ratio (FCR, kg feed/kg gain—lower values indicate better efficiency) and (**B**) average daily gain (ADG, kg/day) for different nitrogen source and carbohydrate fraction combinations. Data represent pooled estimates from 67 performance trials including cattle (*n* = 45), sheep (*n* = 18), and goats *(n* = 4), with total of 1847 animals evaluated. Statistical significance determined using mixed-effects models accounting for species, initial body weight, and trial duration, with Tukey’s post hoc comparisons (*p* < 0.05). CHO = carbohydrates; CRU = controlled-release urea; AA = amino acids; NFC = non-fibrous carbohydrates.

Data from 67 performance trials. Figure 9A demonstrates that optimal synchronization strategies achieved significant FCR improvements: CRU + slowly degrading CHO (5.82 ± 0.42, representing 15% improvement vs. baseline), bypass AA + fibrous CHO (5.95 ± 0.38, 13% improvement), and true protein + slowly degrading CHO (6.15 ± 0.35, 10% improvement).

Baseline (conventional urea + rapidly degrading CHO) showed FCR of 6.85 ± 0.52. Poorest performance observed with conventional urea + fibrous CHO (7.68 ± 0.61). The baseline reference, conventional urea with rapidly degrading CHO, showed an FCR of 6.85 (95% CI: 6.32–7.38), while the poorest-performing combination, conventional urea with fibrous CHO, reached 7.68 (95% CI: 7.07–8.29), representing a 12% reduction in feed efficiency relative to the baseline.

These FCR improvements translate into substantial economic benefits. For example, in a typical feedlot steer (400 kg entry weight, 150 kg gain, 2.5 kg/day ADG), a 15% FCR improvement reduces feed consumption by approximately 135 kg per animal, equivalent to savings of $18–27 at current feed costs.

Average daily gain (ADG) improvements mirrored feed conversion enhancements, with optimal nitrogen–carbohydrate combinations increasing gains by 12–18%. Figure 9B shows corresponding ADG responses, with best combinations yielding 12–18% higher daily gains: CRU + slow CHO achieved 1.42 ± 0.12 kg/day (18% improvement), bypass AA + fibrous CHO reached 1.38 ± 0.11 kg/day (15% improvement), while baseline produced 1.20 ± 0.09 kg/day.

The baseline, conventional urea with rapidly degrading carbohydrates, achieved 1.20 kg/day (95% CI: 1.11–1.29), whereas the poorest-performing combination, conventional urea with fibrous carbohydrates, reached 0.98 kg/day (95% CI: 0.87–1.09), 18% below baseline. These ADG improvements shorten days on feed and reduce production costs. For a 150 kg gain target, an 18% ADG increase (from 1.20 to 1.42 kg/day) reduces time to market by approximately 23 days, saving $46–69 per animal in operating expenses.

Twenty-eight dairy cow studies assessed the effects of N–CHO synchronization on lactational performance.

1. Milk Yield: Optimal synchronization increased milk production by 1.8–3.2 kg/day. Specifically, CRU combined with slowly degrading carbohydrates raised milk yield by 2.8 kg/day (*p* < 0.01) in high-producing cows (>35 kg/day baseline).
2. Milk Protein: True milk protein yield improved by 45–78 g/day with optimized synchronization. Supplementation with bypass amino acids, particularly methionine and lysine, increased milk protein concentration by 0.08–0.15 percentage points.
3. Milk Urea Nitrogen (MUN): MUN decreased by 2.1–4.3 mg/dL under optimal synchronization, reflecting enhanced nitrogen capture efficiency. This reduction is associated with lower urinary nitrogen excretion and decreased environmental nitrogen loading.

## 4. Discussion

The superior performance of CRU combined with slowly degrading carbohydrates is explained by improved synchrony between nitrogen and energy availability to ruminal microorganisms [2,4]. Efficient microbial protein synthesis requires the simultaneous supply of fermentable energy and nitrogen sources [43,44,45,46]. Conventional urea releases ammonia rapidly (1–2 h), creating a mismatch with slowly fermentable substrates, whose peak fermentation occurs later (6–12 h), leading to ammonia losses via absorption and urinary excretion [5,47,48,49,50]. This is particularly limiting for fiber-degrading bacteria, which have slower growth rates and benefit from prolonged nitrogen availability [33,49].

CRU technologies, using lipid, polymer, or polysaccharide matrices, extend ammonia release for 8–16 h, improving synchrony with structural carbohydrate degradation [8,9,10,11,12,13,46,47,48,49,50,51,52,53,54]. This controlled release enhances microbial nitrogen capture, increasing microbial protein synthesis and reducing urinary nitrogen losses [7,51]. Studies consistently show that protected urea improves ruminal nitrogen use efficiency, stabilizes ruminal pH, sustains microbial growth, and enhances fiber digestion, while mitigating environmental nitrogen losses [8,9,10,11,12,13,55,56,57,58,59,60,61,62,63].

Netto et al. [10] demonstrated that including up to 30 g/kg of carnauba wax–microencapsulated urea in sheep diets improved dry matter intake, nutrient digestibility, nitrogen balance, and animal performance, while reducing blood and urinary urea, indicating safer and more efficient use of NPN. Similarly, Melo et al. [9] showed that polysaccharide-matrix CRU improved ruminal fermentation, nitrogen metabolism, digestibility, and microbial protein synthesis by synchronizing urea release with structural carbohydrate degradation [8]. Calcium alginate–encapsulated urea at 1% of dietary DM was identified as an effective and safe CRU source [9,10].

Geron et al. [51] reported improved nitrogen balance and wool production with coated urea, while Da Silva et al. [11] found beeswax-based CRU microspheres without sulfur to provide superior encapsulation efficiency and metabolic responses. In low-quality forage diets dominated by slowly fermentable carbohydrates, conventional urea shows poor synchrony, making bypass amino acids or true protein sources more suitable to support metabolizable protein supply and reduce nitrogen losses [64,65,66,67,68,69,70,71,72,73]. Advances in bypass AA technologies further enhance post-ruminal amino acid availability and nitrogen efficiency [14,74,75,76,77,78,79,80,81,82].

Rumen-protected methionine is well established as a limiting amino acid in ruminant diets, with supplementation increasing milk protein yield by 8–12% in metabolizable protein–deficient diets and showing intestinal digestibility between 65% and 88%, depending on protection technology [83,84,85]. Similar responses have been reported for rumen-protected lysine, particularly when combined with methionine, resulting in up to 14% higher milk protein yield and effective ruminal escape and intestinal digestibility [86,87]. In sheep, wax-based encapsulated escape lysine, especially when combined with tannins, improved nutrient utilization and feeding efficiency without adverse metabolic effects [19].

Multi–protected amino acid strategies combined with slow-release urea further enhance nitrogen utilization and productive performance [88]. Proper nutrient synchrony is closely linked to ruminal pH stability, which is critical for fibrolytic activity and fiber digestion [56,89,90,91,92,93,94]. True protein sources provide adaptive nitrogen release due to heterogeneous degradation kinetics [14,71,74]. When nitrogen release is synchronized with carbohydrate fermentation, microbial efficiency, fiber digestibility, and N retention improve, whereas asynchronous systems increase ruminal ammonia and N losses [95,96,97,98,99,100,101,102,103,104,105,106,107,108].

Emerging evidence indicates that nitrogen–carbohydrate (N–CHO) synchrony affects not only microbial efficiency but also rumen ecosystem stability, increasing microbial diversity, functional redundancy, and resilience to dietary fluctuations, thereby improving nitrogen use efficiency and reducing environmental losses and greenhouse gas emissions [109,110,111]. Bypass amino acids are particularly effective in fibrous diets, as fiber-degrading bacteria exhibit slower growth rates and benefit from sustained post-ruminal amino acid supply [16,17,33,38]. Acetate-dominated fermentation patterns in forage diets may further enhance nitrogen retention through altered metabolic partitioning [78,89,93].

In high-concentrate diets, conventional urea aligns well with rapid carbohydrate fermentation, although partial replacement with CRU (25–50%) improves pH stability and late-phase nitrogen availability [3,6,58,67,91,112]. In forage-based systems, extended fiber fermentation favors CRU or slowly degradable proteins, improving NDF digestibility, microbial protein synthesis, and animal performance [7,20,43,50,51,55,113,114]. Low-quality tropical forages show particularly strong responses to CRU supplementation, markedly enhancing intake, digestibility, and growth by sustaining nitrogen supply throughout prolonged fermentation periods [43,44,51,90].

The combination of CRU with rumen-undegradable protein (RUP) provides synergistic benefits in tropical systems by supporting ruminal microbial growth while supplying post-ruminal amino acids for tissue protein synthesis [90,115]. For low-quality tropical forages, combining CRU with high-RUP supplements effectively corrects both ruminal and post-ruminal protein deficiencies. Integration of nutrient synchronization concepts with precision feeding and dynamic modeling allows optimization of nitrogen supplementation based on carbohydrate profiles, passage rate, and production stage, improving economic and environmental efficiency [101,102,116,117,118,119].

Improved nitrogen efficiency enables reductions in dietary crude protein without performance losses, offsetting CRU costs and increasing net returns in feedlot and dairy systems [7,67,105,107,120,121,122,123]. Additionally, optimized N–CHO synchrony reduces nitrogen excretion, mitigating regulatory costs, ammonia and nitrous oxide emissions, nutrient losses, and overall environmental impacts while improving resource-use efficiency and sustainability in ruminant production systems [72,96,98,99,124,125,126].

## 5. Limitations and Knowledge Gaps

Several limitations must be considered when interpreting studies on N–CHO synchronization in ruminant nutrition. A major constraint is the heterogeneity of carbohydrate characterization methods, ranging from proximate analysis to enzymatic and detergent fiber fractionation, which complicates cross-study comparisons and may mask interaction effects between nitrogen and carbohydrate degradability [21,127,128,129]. Discrepancies between in vitro and in vivo approaches further limit extrapolation, as in vitro systems cannot fully reproduce rumen dynamics, including pH fluctuations, passage rate, and microbial adaptation [65,130,131,132]. In addition, the predominance of short-term trials restricts understanding of long-term microbial and metabolic adaptations [133,134].

Most studies also evaluate N–CHO synchronization in isolation, neglecting interactions with lipids, minerals, feed additives, and processing methods that can alter rumen fermentation and nutrient kinetics [53,135,136]. Future research should adopt mechanistic approaches using stable isotopes and meta-omics to quantify nitrogen flows and microbial responses [106,108,109], coupled with dynamic modeling frameworks integrated into ration formulation systems [117,118,137,138]. Greater emphasis is needed on genotype-specific responses, tropical and extensive systems, small ruminants, and environmental stressors, which remain underrepresented in the literature [43,50,90,139,140,141,142,143,144].

## 6. Conclusions

This systematic review provides strong evidence that interactions between nitrogen sources and carbohydrate fractions are key determinants of rumen metabolism, nitrogen use efficiency, and animal performance. From 164 screened studies, 89 met the inclusion criteria, demonstrating that optimal synchronization is source- and context-dependent. Controlled-release urea (CRU) combined with slowly degrading carbohydrates emerged as the most effective strategy, markedly improving microbial protein synthesis, nitrogen efficiency, and feed conversion. Bypass amino acids were particularly effective in high-forage systems, while true protein sources showed consistent, adaptable performance across carbohydrate fractions. Conventional urea was deemed only suitable for high-concentrate diets.

Evidence quality was high for conventional urea and true protein interactions and moderate for CRU and bypass amino acids due to variability in technologies. Economically, optimized synchronization generated significant returns in feedlot and dairy systems, while environmentally it reduced nitrogen losses and emissions. Overall, the findings support a paradigm shift from crude protein–based feeding toward mechanistic nitrogen–carbohydrate synchronization to enhance productivity, profitability, and sustainability in ruminant systems.

## Figures and Tables

**Table 1:** Optimal Synchronization Strategies.

Nitrogen Source	Optimal Carbohydrate Fraction	MPS Improvement (%)	NUE Improvement (%)	Quality of Evidence
CRU	Slowly degrading soluble CHO	28.3 ± 4.2	24.6 ± 3.8	High
Conventional urea	Rapidly degrading soluble CHO	15.8 ± 3.6	12.3 ± 4.1	High
True protein	Mixed fractions (NFC + Fiber)	19.2 ± 2.8	18.9 ± 3.2	High
Bypass AA	Fibrous CHO (NDF)	22.4 ± 4.5	20.1 ± 4.8	Moderate
Urea + bypass AA	Van Soest fractions (balanced)	25.7 ± 3.9	22.8 ± 3.6	Moderate
CRU	Slowly degrading soluble CHO	28.3 ± 4.2	24.6 ± 3.8	High
Conventional Urea	Rapidly degrading soluble CHO	15.8 ± 3.6	12.3 ± 4.1	High

where AA = amino acids; CHO = carbohydrates; CRU = controlled-Release Urea; MPS = microbial protein synthesis; NUE = nitrogen utilization efficiency; NFC = non-fibrous carbohydrates; NDF = neutral detergent fiber.

**Table 2:** Integration findings from 47 in vitro studies and 38 in vivo trials to provide a comprehensive assessment of nitrogen–carbohydrate synchronization.

Parameter	In Vitro Typical Response	In Vivo Typical Response	Conversion Factor/Considerations
Microbial protein synthesis	+25–35% (g/kg OM fermented)	+15–22% (g/kg OM digested)	In vivo responses are attenuated by passage rate, nitrogen recycling, and metabolic regulation
Ammonia-N concentration	−30–45% reduction with CRU vs. urea	−18–28% reduction with CRU vs. urea	In vivo nitrogen recycling (40–60 g N/day via saliva) buffers ammonia fluctuations
VFA production	+12–18% increase	+8–14% increase	In vivo absorption kinetics and hepatic metabolism moderate VFA accumulation
Nitrogen use efficiency	+28–40% improvement	+16–25% improvement	Post-ruminal efficiency (65–75%) and endogenous losses reduce whole-animal response

## Data Availability

All data extracted for this systematic review are available from the corresponding author upon reasonable request.

## Supplementary Materials

The following supporting information can be downloaded at: https://www.mdpi.com/article/10.3390/ani16020239/s1. Supplementary Tables from Zenodo in link https://doi.org/10.5281/zenodo.17608529: accessed on 14 November 2025. Table S1. CRU_Studies Description of the variables extracted from the studies for conducting the controlled release urea with different animal species (cattle, dairy cows, sheep, beef cattle); Table S2. Urea_Synchronization_Studies: Description of the variables extracted from the studies for conducting the carbohydrate type, synchronization, MPS and NUE changes with different animal species (cattle, dairy cows, sheep, beef cattle); Table S3. Description of the variables extracted from the studies for conducting the protein source × carbohydrate relationship on improvement in MPS (%), and key outcomes with different animal species (cattle, dairy cows, beef cattle); Table S4. Description of the variables extracted from the studies for conducting the by-pass AAs relationship on improvement in NUE (%), and key outcomes with different animal species (cattle, dairy cows, sheep, beef cattle); Table S4. Description of the variables extracted from the studies for conducting the nitrogen source on FCR (kg/kg) and ADG (kg/day) improvement (%), NUE (%), and urinary N reduction (%) with different animal species (cattle, dairy cows, sheep, beef cattle).

## Abbreviations

The following abbreviations are used in this manuscript:

AA	amino acids
ADG	average daily gain
A:P	Acetate: propionate ratio
CHO	carbohydrates
CRU	controlled-release urea
DM	Dry matter
FCR	feed conversion ratio
MPS	microbial protein synthesis
N	Nitrogen
NFC	non-fibrous carbohydrates
NPN	non-protein nitrogen
NUE	nitrogen utilization efficiency
SRU	slow-release urea
VFA	Volatile fatty acid
